# Dopamine Receptors Antagonistically Regulate Behavioral Choice between Conflicting Alternatives in *C. elegans*

**DOI:** 10.1371/journal.pone.0115985

**Published:** 2014-12-23

**Authors:** Daoyong Wang, Yonglin Yu, Yinxia Li, Yang Wang, Dayong Wang

**Affiliations:** Key Laboratory of Developmental Genes and Human Disease in Ministry of Education, Medical School of Southeast University, Nanjing, 210009, China; Inserm U869, France

## Abstract

*Caenorhabditis elegans* is a useful model to study the neuronal or molecular basis for behavioral choice, a specific form of decision-making. Although it has been implied that both D1-like and D2-like dopamine receptors may contribute to the control of decision-making in mammals, the genetic interactions between D1-like and D2-like dopamine receptors in regulating decision-making are still largely unclear. In the present study, we investigated the molecular control of behavioral choice between conflicting alternatives (diacetyl and Cu^2+^) by D1-like and D2-like dopamine receptors and their possible genetic interactions with *C. elegans* as the assay system. In the behavioral choice assay system, mutation of *dop-1* gene encoding D1-like dopamine receptor resulted in the enhanced tendency to cross the Cu^2+^ barrier compared with wild-type. In contrast, mutations of *dop-2* or *dop-3* gene encoding D2-like dopamine receptor caused the weak tendency to cross the Cu^2+^ barrier compared with wild-type. During the control of behavioral choice, DOP-3 antagonistically regulated the function of DOP-1. The behavioral choice phenotype of *dop-2; dop-1dop-3* triple mutant further confirmed the possible antagonistic function of D2-like dopamine receptor on D1-like dopamine receptor in regulating behavioral choice. The genetic assays further demonstrate that DOP-3 might act through Gα_o_ signaling pathway encoded by GOA-1 and EGL-10, and DOP-1 might act through Gα_q_ signaling pathway encoded by EGL-30 and EAT-16 to regulate the behavioral choice. DOP-1 might function in cholinergic neurons to regulate the behavioral choice, whereas DOP-3 might function in GABAergic neurons, RIC, and SIA neurons to regulate the behavioral choice. In this study, we provide the genetic evidence to indicate the antagonistic relationship between D1-like dopamine receptor and D2-like dopamine receptor in regulating the decision-making of animals. Our data will be useful for understanding the complex functions of dopamine receptors in regulating decision-making in animals.

## Introduction

Dopamine regulates a variety of behavioral activities in both vertebrates and invertebrates. In mammals, dopamine can act through five receptors that are grouped into several classes. D1-like and D2-like dopamine receptors usually have antagonistic effects on behaviors in mammals [1]. In *Caenorhabditis elegans*, there are four dopamine receptors: DOP-1, DOP-2, DOP-3, and DOP-4 [2]. DOP-1 and DOP-4 are D1-like dopamine receptors. DOP-2 and DOP-3 are D2-like dopamine receptors. DOP-4 is unique in invertebrate and distinct from mammalian D1-like dopamine receptors [3]. Previous study has demonstrated that DOP-1 and DOP-3 had antagonistic effects on basal slowing response in *C. elegans* [2]. DOP-1 and DOP-3 could further activate antagonistic Gα_q_ and Gα_o_ signaling pathways to regulate the basal slowing response [2].

*C. elegans* is a useful model system to study the neuronal or molecular basis for behaviors including decision-making [4]-[6]. In *C. elegans*, among the several forms of decision-making, behavioral choice between conflicting alternatives (diacetyl and Cu^2+^) can reflect the effects of multiple stimuli (attractant versus aversive stimuli) on behavioral plasticity in animals [7]–[9]. Nematodes normally show being attracted to attractants such as diacetyl which is sensed by AWA sensory neurons [10], but will avoid aversive cues such as Cu^2+^ ion which is sensed by ADL/ASH sensory neurons [11]. For the molecular mechanism of behavioral choice between conflicting alternatives, some signaling pathways have been raised to have important roles. HEN-1 (hesitation behavior) was first identified to play an important role in behavioral choice control in AIY interneurons [7]. HEN-1 and SCD-2 (suppressor of constitutive dauer formation), a target of FSN-1 (F-box synaptic protein), may function in the same genetic pathway to regulate behavioral choice [9], [12]. GCY-28 (guanylyl cylase)/CNG-1 (cyclic nucleotide gated channel) functioned in a parallel pathway in AIA interneurons with HEN-1/SCD-2 to regulate behavioral choice [9]. The insulin signaling pathway including *daf-16*, *daf-2*, and *daf-18* was also shown to participate in the control of behavioral choice [13].

Decision-making is a complex cognitive process that is found to be impaired in a number of psychiatric diseases. Previous studies have demonstrated that both D1-like and D2-like dopamine receptors may contribute to the decision-making impairments in human and animals [14]–[18]. However, the genetic interactions between D1-like and D2-like dopamine receptors in regulating decision-making are still largely unclear. In the present study, we investigated the molecular control of behavioral choice by D1-like and D2-like dopamine receptors and their possible genetic interactions with *C. elegans* as the *in vivo* assay system. Our data suggest the antagonistic functions between D1-like and D2-like dopamine receptors in regulating the behavioral choice in nematodes. Our study will be useful for understanding the important function of dopamine signaling in regulating the decision-making in animals.

## Results

### Effects of mutations of genes encoding D1-like dopamine receptors on behavioral choice

**Figure 1:**
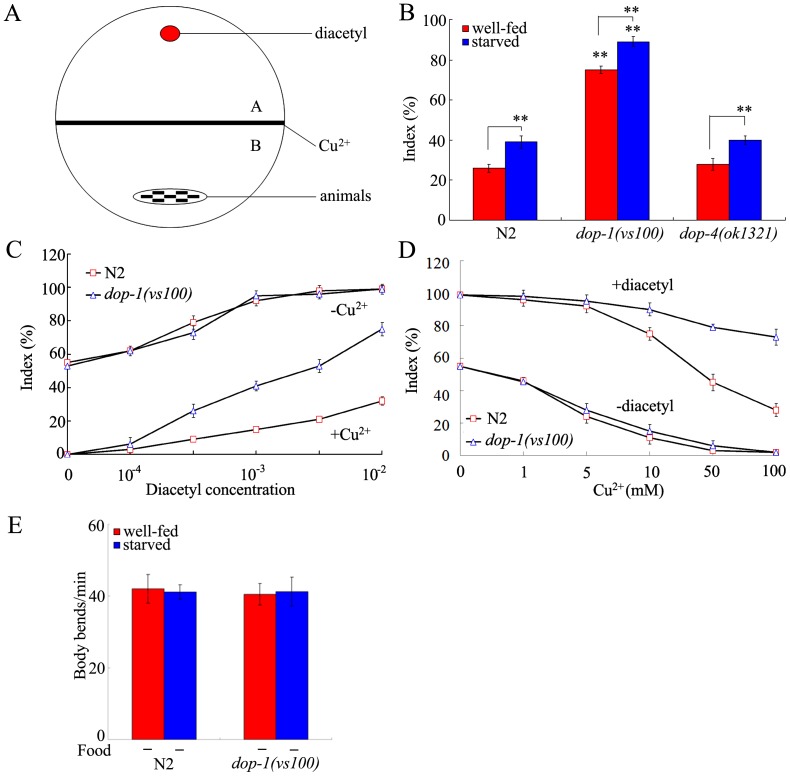
Effects of D1-like dopamine receptor on behavioral choice between conflicting alternatives. (A) Assay model for the behavioral choice between conflicting alternatives. (B) Phenotypes of *dop-1* and *dop-4* mutants in the interaction assay under the well-fed or starved condition. In the assay system, the Cu^2+^ ion concentration was 100 mM, and the diacetyl concentration was 10^−2^. (C) Dose-response curves of wild-type N2 and *dop-1* mutants to diacetyl with (+) or without (-) 100 mM of Cu^2+^ ion. Differences between groups were determined using two-way ANOVA. (D) Dose-response curves of wild-type N2 and *dop-1* mutants to Cu^2+^ ion with (+) or without (-) 10^−2^ of diacetyl. Differences between groups were determined using two-way ANOVA. (E) Locomotion behavior of wild-type N2 and *dop-1* mutants in the absence (-) of food under well-fed or starved condition. Locomotion behavior was assessed by the body bend. Bars represent mean ± S.E.M. **P <0.01 *vs* N2 (if not specially indicated).

In *C. elegans*, D1-like dopamine receptors contain DOP-1 and DOP-4. In the behavioral choice assay system (Fig. 1A), the *dop-4* loss-of-function mutant (*dop-4(ok1321)*) showed the similar tendency to cross the Cu^2+^ barrier to that of wild-type under both the well-fed and the starved conditions (Fig. 1B). In contrast, the *dop-1* loss-of-function mutant (*dop-1(vs100)*) exhibited the enhanced tendency to cross the Cu^2+^ barrier compared with wild-type under the well-fed conditions (Fig. 1B). In the assay system, when either diacetyl or Cu^2+^ ion was presented, index of *dop-1(vs100)* mutant was similar to that of wild-type animals (Fig. 1C and 1D), implying that the observed deficit in behavioral choice in *dop-1* mutant is not due to the abnormality of chemotaxis to diacetyl or avoidance of Cu^2+^. However, when both Cu^2+^ ion and diacetyl were presented in the assay system, index of *dop-1(vs100)* mutant was higher than that of wild-type animals (Fig. 1C and 1D). Thus, the inhibition of Cu^2+^ avoidance by diacetyl may be stronger than the inhibition of diacetyl chemotaxis by Cu^2+^ ion in *dop-1* mutants.

Due to the sensation of starvation, starved wild-type nematodes show the higher index of behavioral choice than well-fed wild-type nematodes [7]. Like wild-type animals, *dop-1(vs100)* mutant changed their behavioral choice after starvation (Fig. 1B), demonstrating that the *dop-1* mutant can sense the starvation like wild-type nematodes. In the behavioral choice assay system, if the examined nematodes are abnormal in locomotion behavior, the obtained index of behavioral choice may not be able to reflect the real ability of behavioral choice for the nematodes. In this study, we used the body bend to reflect the state of locomotion behavior of nematodes. Both the well-fed and the starved *dop-1(vs100)* mutants showed the similar body bends to those of wild-type in the NGM plates without food (Fig. 1E), suggesting the normal locomotion behavior of *dop-1* mutant in the behavioral choice assay system. These data suggest that *dop-1* mutant does have deficits in behavioral choice between conflicting alternatives.

### Effects of mutations of genes encoding D2-like dopamine receptors on behavioral choice

**Figure 2:**
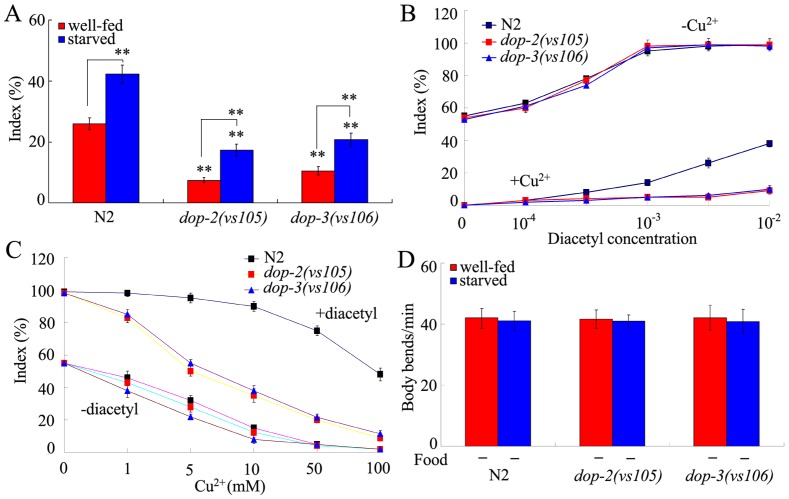
Effects of D2-like dopamine receptor on behavioral choice between conflicting alternatives. (A) Phenotypes of *dop-2* and *dop-3* mutants in the interaction assay under the well-fed or starved condition. In the assay system, the Cu^2+^ ion concentration was 100 mM, and the diacetyl concentration was 10^−2^. (B) Dose-response curves of wild-type N2 and mutants to diacetyl with (+) or without (-) 100 mM of Cu^2+^ ion. Differences between groups were determined using two-way ANOVA. (C) Dose-response curves of wild-type N2 and mutants to Cu^2+^ ion with (+) or without (-) 10^−2^ of diacetyl. Differences between groups were determined using two-way ANOVA. (D) Locomotion behavior of wild-type N2 and mutants in the absence (-) of food under the well-fed or starved condition. Locomotion behavior was assessed by the body bend. Bars represent mean ± S.E.M. **P <0.01 *vs* N2 (if not specially indicated).

In *C. elegans*, D2-like dopamine receptors contain DOP-2 and DOP-3. In the behavioral choice assay system, both *dop-2* and *dop-3* loss-of-function mutants (*dop-2(vs105)* and *dop-3(vs106)*) showed the weak tendency to cross the Cu^2+^ barrier compared with wild-type under the well-fed conditions (Fig. 2A). In the assay system, index of *dop-2(vs105)* or *dop-3(vs106)* mutant was similar to that of wild-type animals when either diacetyl or Cu^2+^ ion was presented (Fig. 2B and 2C), implying that the observed deficit in behavioral choice in *dop-2* or *dop-3* mutant may be not due to the abnormality in diacetyl chemotaxis or Cu^2+^ avoidance. In the assay system, different from the *dop-1(vs100)* mutant, index of *dop-2(vs105)* or *dop-3(vs106)* mutant was lower than that of wild-type animals when both Cu^2+^ ion and diacetyl were presented in the assay system (Fig. 2B and 2C), implying that the inhibition of diacetyl chemotaxis by Cu^2+^ ion may be stronger than the inhibition of Cu^2+^ avoidance by diacetyl in *dop-2* and *dop-3* mutants.

*dop-2(vs105)* and *dop-3(vs106)* mutants could change their behavioral choice after starvation (Fig. 2A), demonstrating that the *dop-2* and *dop-3* mutants can sense the starvation like wild-type nematodes. The *dop-2(vs105)* and *dop-3(vs106)* mutants also had the normal locomotion behavior in the absence of food under the well-fed or starved condition (Fig. 2D), implying that the observed deficit in behavioral choice in *dop-2* or *dop-3* mutant may be not due to the abnormality in locomotion behavior in the behavioral choice assay system. Thus, both DOP-2 and DOP-3 participate in the control of behavioral choice between conflicting alternatives in nematodes.

### Genetic interactions of D1-like dopamine receptor and D2-like dopamine receptor in regulating behavioral choice

**Figure 3:**
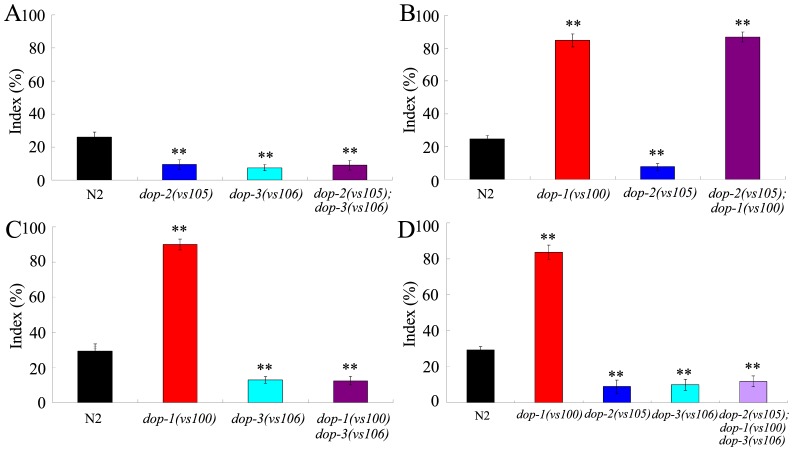
Genetic interactions of D1-like dopamine receptor with D2-like dopamine receptors in regulating behavioral choice between conflicting alternatives. (A) Genetic interaction between DOP-2 and DOP-3 in regulating behavioral choice. (B) Genetic interaction between DOP-1 and DOP-2 in regulating behavioral choice. (C) Genetic interaction between DOP-1 and DOP-3 in regulating behavioral choice. (D) The behavioral choice phenotype of *dop-2; dop-1dop-3* triple mutant. The behavioral choice was examined under the well-fed condition. In the assay system, the Cu^2+^ ion concentration was 100 mM, and the diacetyl concentration was 10^−2^. Bars represent mean ± S.E.M. **P<0.01.

We next examined the genetic interactions between D1-like dopamine receptor and D2-like dopamine receptor in regulating behavioral choice in *C. elegans*. The double mutant of *dop-2(vs105); dop-3(vs106)* showed the similar behavioral choice phenotype to that of *dop-2(vs105)* or *dop-3(vs106)* mutant (Fig. 3A), implying that these two D2-like dopamine receptor genes may function in the same genetic pathway to regulate the behavioral choice.

The double mutant of *dop-2(vs105); dop-1(vs100)* showed the similar behavioral choice phenotype to that of *dop-1(vs100)* mutant (Fig. 3B). Different from the behavioral choice phenotype of *dop-2(vs105); dop-1(vs100)* mutant, the *dop-1(vs100)dop-3(vs106)* exhibited the similar behavioral choice phenotype to that of *dop-3(vs106)* mutant (Fig. 3C). These data suggest that the *dop-3* mutation, but not the *dop-2* mutation, can reverse the functions of *dop-1* mutation in regulating behavioral choice.

The triple mutant of *dop-2(vs105); dop-1(vs100)dop-3(vs106)* showed the similar behavioral choice phenotype to that in *dop-2(vs105)* or *dop-3(vs106)* mutant, and not exhibited the similar behavioral choice phenotype to that in *dop-1(vs100)* mutant (Fig. 3D). These data imply that, in the behavioral choice control of nematodes without functions of dopamine receptors, the effects from mutation of genes encoding D1-like dopamine receptors may be suppressed by the mutation of genes encoding D2-like dopamine receptors.

### Effects of mutations of genes encoding Gα_q_ signaling pathway on behavioral choice

**Figure 4:**
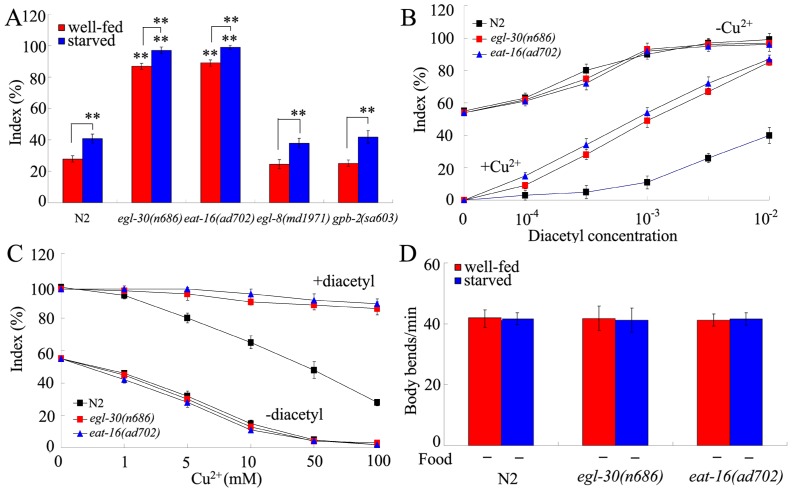
Roles of Gα_q_ signaling pathway in regulating behavioral choice between conflicting alternatives. (A) Phenotypes of Gα_q_ signaling pathway mutants in the interaction assay under the well-fed or starved condition. In the assay system, the Cu^2+^ ion concentration was 100 mM, and the diacetyl concentration was 10^−2^. (B) Dose-response curves of wild-type N2 and mutants to diacetyl with (+) or without (-) 100 mM of Cu^2+^ ion. Differences between groups were determined using two-way ANOVA. (C) Dose-response curves of wild-type N2 and mutants to Cu^2+^ ion with (+) or without (-) 10^−2^ of diacetyl. Differences between groups were determined using two-way ANOVA. (D) Locomotion behavior of wild-type N2 and mutants in the absence (-) of food under the well-fed or starved condition. Locomotion behavior was assessed by the body bend. Bars represent mean ± S.E.M. **P<0.01 *vs* N2 (if not specially indicated).

Previous study has suggested that D1-like dopamine receptors can activate Gα_q_ signaling pathway in cells [2]. In *C. elegans*, the Gα_q_ signaling pathway contains *egl-30*, *eat-16*, *egl-8*, and *gpb-2* genes. In the behavioral choice assay system, the *egl-8(md197)* and *gpb-2(sa603)* mutants had the similar tendency to cross the Cu^2+^ barrier to that of wild-type under both the well-fed and the starved conditions (Fig. 4A). In contrast, the *egl-30(n686)* and *eat-16(ad702)* mutants showed the enhanced tendency to cross the Cu^2+^ barrier compared with wild-type under both the well-fed and the starved conditions (Fig. 4A). In the assay system, index of *egl-30(n686)* or *eat-16(ad702)* mutant was similar to that of wild-type animals when either diacetyl or Cu^2+^ ion was presented (Fig. 4B and 4C), implying that the observed deficit in behavioral choice in *egl-30* or *eat-16* mutant may be not due to the abnormality in diacetyl chemotaxis or Cu^2+^ avoidance. In the assay system, index of *egl-30(n686)* or *eat-16(ad702))* mutant was higher than that of wild-type animals when both Cu^2+^ ion and diacetyl were presented in the assay system (Fig. 4B and 4C), suggesting that the inhibition of Cu^2+^ avoidance by diacetyl may be stronger than the inhibition of diacetyl chemotaxis by Cu^2+^ ion in *egl-30* and *eat-16* mutants. Both *egl-30(n686)* and *eat-16(ad702)* mutants were normal in the sensation of starvation (Fig. 4A), and showed the normal locomotion behavior in the absence of food under well-fed or starved condition (Fig. 4D). These results suggest that, among the members of Gα_q_ signaling pathway, EGL-30 and EAT-16 regulate the behavioral choice between conflicting alternatives in nematodes.

### Effects of mutations of genes encoding Gα_o_ signaling pathway on behavioral choice

**Figure 5:**
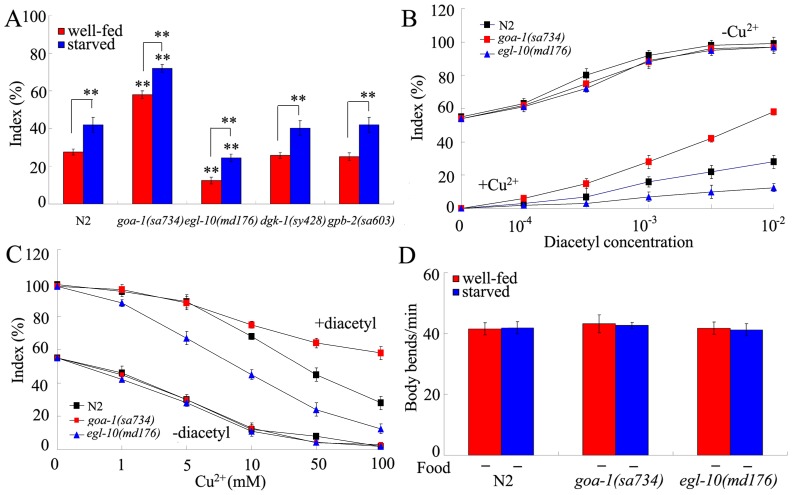
Roles of Gα_o_ signaling pathway in regulating behavioral choice between conflicting alternatives. (A) Phenotypes of Gα_o_ signaling pathway mutants in the interaction assay under the well-fed or starved condition. In the assay system, the Cu^2+^ ion concentration was 100 mM, and the diacetyl concentration was 10^−2^. (B) Dose-response curves of wild-type N2 and mutants to diacetyl with (+) or without (-) 100 mM of Cu^2+^ ion. Differences between groups were determined using two-way ANOVA. (C) Dose-response curves of wild-type N2 and mutants to Cu^2+^ ion with (+) or without (-) 10^−2^ of diacetyl. Differences between groups were determined using two-way ANOVA. (D) Locomotion behavior of wild-type N2 and mutants in the absence (-) of food under the well-fed or starved condition. Locomotion behavior was assessed by the body bend. Bars represent mean ± S.E.M. **P<0.01 *vs* N2 (if not specially indicated).

Previous study has suggested that D2-like dopamine receptors can activate Gα_o_ signaling pathway in cells [2]. In *C. elegans*, besides the *gpb-2* gene, the Gα_o_ signaling pathway contains *goa-1*, *egl-10*, and *dgk-1* genes. In the behavioral choice assay system, the *dgk-1(sy428)* and *gpb-2(sa603)* mutants exhibited the similar tendency to cross the Cu^2+^ barrier to that of wild-type under both the well-fed and the starved conditions (Fig. 5A). In contrast, the *goa-1(sa723)* mutant showed the enhanced tendency to cross the Cu^2+^ barrier, and the *egl-10(md176)* mutant exhibited the weak tendency to cross the Cu^2+^ barrier compared with wild-type under both the well-fed and the starved conditions (Fig. 5A). In the assay system, indexes of *goa-1(sa723)* and *egl-10(md176)* mutants were similar to that of wild-type animals when either diacetyl or Cu^2+^ ion was presented (Fig. 5B and 5C), implying that the observed deficit in behavioral choice in *goa-1* or *egl-10* mutant may be not due to the abnormality in diacetyl chemotaxis or Cu^2+^ avoidance. In the assay system, the index of *goa-1(sa723)* mutant was higher than that of wild-type animals and the index of *egl-10(md176)* mutant was lower than that of wild-type animals when both Cu^2+^ ion and diacetyl were presented in the assay system (Fig. 5B and 5C). That is, the inhibition of Cu^2+^ avoidance by diacetyl may be stronger than the inhibition of diacetyl chemotaxis by Cu^2+^ ion in *goa-1* mutant, and the inhibition of diacetyl chemotaxis by Cu^2+^ ion may be stronger than the inhibition of Cu^2+^ avoidance by diacetyl in *egl-10* mutant. Both *goa-1(sa723)* and *egl-10(md176)* mutants could normally sense the starvation (Fig. 5A), and showed the normal locomotion behavior in the absence of food under the well-fed or the starved condition (Fig. 5D). Therefore, among the members of Gα_o_ signaling pathway, GOA-1 and EGL-10 are involved in the control of behavioral choice between conflicting alternatives in nematodes.

### Genetic interactions of DOP-1 with EGL-30 or EAT-16 in regulating behavioral choice

**Figure 6:**
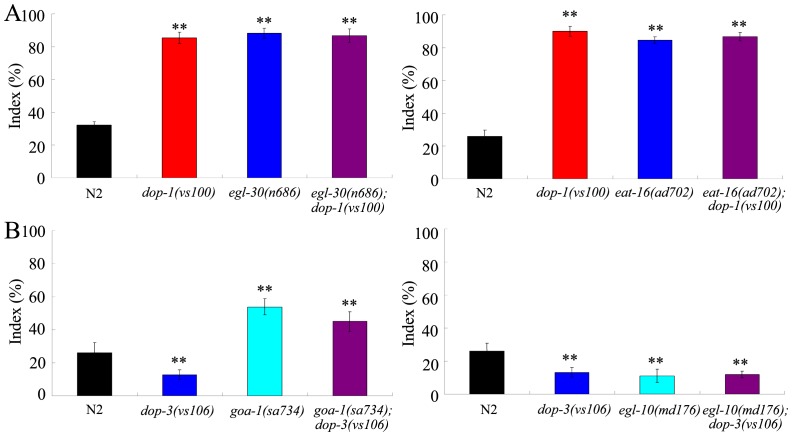
Genetic interactions of dopamine receptors with G-protein signaling pathways in regulating behavioral choice between conflicting alternatives. (A) Genetic interactions of DOP-1 with Gα_q_ signaling pathway in regulating behavioral choice between conflicting alternatives. (B) Genetic interactions of DOP-3 with Gα_o_ signaling pathway in regulating behavioral choice between conflicting alternatives. The behavioral choice was examined under the well-fed condition. In the assay system, the Cu^2+^ ion concentration was 100 mM, and the diacetyl concentration was 10^−2^. Bars represent mean ± S.E.M. **P<0.01.

We next examined the genetic interactions of *dop-1* gene with *egl-30* or *eat-16* gene in regulating the behavioral choice. In the behavioral choice assay system, all of the *dop-1(vs100)*, *egl-30(n686)*, and *eat-16(ad702)* mutants had the enhanced tendency to cross the Cu^2+^ barrier. The behavioral choice phenotype of *egl-30(n686); dop-1(vs100)* double mutant was similar to that in *dop-1(vs100)* or *egl-30(n686)* mutant (Fig. 6A). Moreover, the behavioral choice phenotype of *eat-16(ad702); dop-1(vs100)* double mutant was similar to that in *dop-1(vs100)* or *eat-16(ad702)* mutant (Fig. 6A). Therefore, DOP-1 may act in the same genetic pathway with EGL-30 and EAT-16 to regulate the behavioral choice between conflicting alternatives in nematodes.

### Genetic interactions of DOP-3 with GOA-1 or EGL-10 in regulating behavioral choice

We further examined the genetic interactions of *dop-3* gene with *goa-1* or *egl-10* gene in regulating the behavioral choice. In the behavioral choice assay system, the *dop-3(vs106)* and *egl-10(md176)* mutants had the weak tendency to cross the Cu^2+^ barrier, and the *goa-1(sa734)* mutant had the enhanced tendency to cross the Cu^2+^ barrier. The behavioral choice phenotype of *goa-1(sa734); dop-3(vs106)* double mutant was similar to that in *goa-1(sa734)* mutant (Fig. 6B). That is, mutation of *goa-1* gene could reverse the behavioral choice phenotype caused by mutation of *dop-3* gene. Moreover, the behavioral choice phenotype of *egl-10(md176); dop-3(vs106)* double mutant was similar to that in *dop-3(vs106)* or *egl-10(md176)* mutant (Fig. 6B). Therefore, DOP-3 may act in the same genetic pathway with GOA-1 and EGL-10 to regulate behavioral choice between conflicting alternatives in nematodes.

### Neuronal circuit of DOP-1 or DOP-3 in regulating behavioral choice

**Figure 7:**
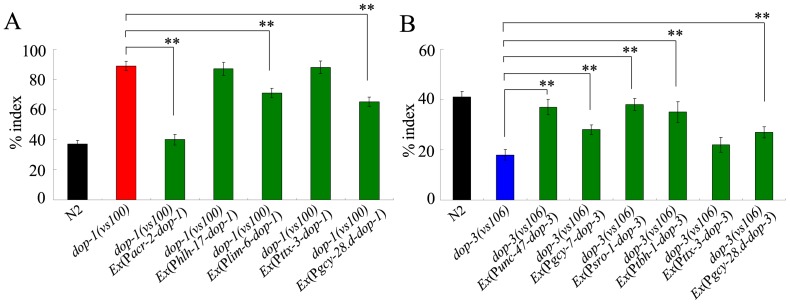
Neuron-specific activity of DOP-1 or DOP-3 in regulating behavioral choice. (A) Neuron-specific activity of DOP-1 in regulating behavioral choice. (B) Neuron-specific activity of DOP-3 in regulating behavioral choice. The behavioral choice was examined under the well-fed condition. In the assay system, the Cu^2+^ ion concentration was 100 mM, and the diacetyl concentration was 10^−2^. In the rescue experiments, *hlh-17* promoter was used for glia-specific expression, *unc-47* promoter was used for GABAergic neurons-specific expression, *acr-2* promoter was used for cholinergic neuron-specific expression, *gcy-28.d* promoter was used for AIA-specific expression, *ttx-3* promoter was used for AIY-specific expression, *tbh-1* promoter was used for RIC-specific expression, *gcy-7* promoter was used for ASE-specific expression, *sro-1* promoter was used for expression in SIA neurons, and *lim-6* promoter was used for expression in RIS neurons. Bars represent mean ± S.E.M. **P<0.01.

In *C. elegans*, DOP-1 was expressed in sheath cells, and some neurons in the head including RIS interneuron [2], [19], and DOP-3 was expressed in some neurons in the head including ASE, SIA, and RIC neurons, mechanosensory neurons, and body-wall muscles [2], [20]-[21]. DOP-1 was specially expressed in the cholinergic neurons, whereas DOP-3 was strongly expressed in GABAergic neurons [2]. Both DOP-1 and DOP-3 were not expressed in command interneurons that affect the locomotion behavior [2]. The expression of DOP-1 was not overlapped with that of DOP-3 [2]. Our results showed that, in *dop-1(vs100)* mutant, expression of DOP-1 in cholinergic neurons could rescue its deficit in behavioral choice (Fig. 7A). In contrast, expression of DOP-1 in sheath cells did not rescue the deficit in behavioral choice of *dop-1(vs100)* mutant, and expression of DOP-1 in RIS neurons could only moderately rescue the deficit in behavioral choice of *dop-1(vs100)* mutant (Fig. 7A). Moreover, in *dop-3(vs106)* mutant, expression of DOP-3 in GABAergic neurons could rescue its deficit in behavioral choice (Fig. 7B). Interestingly, expression of DOP-3 in RIC or SIA neurons could also rescue the deficit in behavioral choice of *dop-3(vs106)* mutant (Fig. 7B). In contrast, expression of DOP-3 in ASE neurons could only moderately rescue the deficit in behavioral choice of *dop-3(vs106)* mutant (Fig. 7B).

Besides these, we found that expression of DOP-1 or DOP-3 in AIY interneurons did not rescue the deficit in behavioral behavior in the corresponding mutant (Fig. 7). In contrast, expression of DOP-1 or DOP-3 in AIA interneurons could only moderately rescue the deficit in behavioral behavior in the corresponding mutant (Fig. 7). These results suggest that both DOP-1 and DOP-3 may not function in AIY or AIA interneurons to regulate the behavioral choice.

## Discussion

Previous studies have demonstrated that dopamine receptors can regulate several behaviors including the locomotion, food response, enhancement of odor avoidance, mating behavior, and plasticity of mechanosensory response in *C. elegans* [22]–[26]. In the present study, we provide the evidence to further prove the crucial role of both D1-like dopamine receptor (DOP-1) and D2-like dopamine receptors (DOP-2 and DOP-3) in the control of behavioral choice between conflicting alternatives (diacetyl and Cu^2+^). DOP-2 and DOP-3 may function in the same genetic pathway to regulate behavioral choice. The observed deficits in behavioral choice between conflicting alternatives in *dop-1*, *dop-2*, and *dop-3* loss-of-function mutants were not due to the abnormality in sensation of starvation, and locomotion behavior. Our results are consistent to a certain degree with the observed functions of D1-like and D2-like dopamine receptors in regulating decision-making of mammalian animals. In mammals, D1-like and D2-like dopamine receptors may play the prominent roles in regulating decision-making. In contrast, D3-like or D4-like dopamine receptors might only participate in the control of specific forms of decision-making in mammals [27]–[29]. In *C. elegans*, previous studies have further suggested that dopamine receptors can regulate the behavioral plasticity [22]–[23]. Therefore, D1-like and D2-like dopamine receptors can regulate the two steps of informational processing including both the learning and the integration of sensory signals of nematodes.

In the assay system for behavioral choice, we used two stimuli, diacetyl and Cu^2+^. In *C. elegans*, AWA sensory neurons are involved in the sensation of diacetyl, and the ASH and ADL sensory neurons are involved in the sensation of Cu^2+^ ion [10]–[11]. Moreover, some neurotransmitters, including neuropeptide, serotonin, tyramine and octopamine, have been suggested to modulate the ASH-mediated aversive behaviors [30]–[33]. However, our data suggest that mutations of the examined dopamine receptors had the normal chemotaxis to diacetyl and avoidance of Cu^2+^, suggesting that the examined dopamine receptors may be not involved in the control of the chemotaxis to diacetyl or the avoidance of Cu^2+^.

Our data further demonstrate that D1-like and D2-like dopamine receptors antagonistically regulate the behavioral choice. The D2-like dopamine receptor DOP-3 could antagonistically regulate the function of D1-like dopamine receptor DOP-1 in regulating the behavioral choice. The behavioral choice phenotype of *dop-2(vs105); dop-1(vs100)dop-3(vs106)* triple mutant further confirmed the important antagonistic function of D2-like dopamine receptor on D1-like dopamine receptor in regulating the behavioral choice in nematodes. It has been implied that both D1-like and D2-like dopamine receptors may have antagonistic effects on decision-making in mammals. In rats, based on pharmacological manipulations, blockage of D1-like receptors in prefrontal cortex decreased preference for the risky decision-making; whereas blockage of D2-like receptors in prefrontal cortex increased the risky decision-making [15]. In the present study, we provide the direct genetic evidence to indicate the antagonistic relationship between D1-like dopamine receptor and D2-like dopamine receptor in regulating the decision-making of animals. Previous study has also demonstrated that mutation of *dop-1* gene reversed the deficit in basal slowing response of the *dop-3* mutant nematodes [2]. Therefore, the relationship between D1-like dopamine receptor and D2-like dopamine receptor in regulating the functions of nervous system of nematodes may be very complex.

Our data in the present study suggest that the dopamine signaling is involved in the control of behavioral choice in nematodes. However, the behavioral choice in *C. elegans* may be not solely dependent on the dopamine signaling. Other specific neurotransmitter signaling pathways may be also involved in the control of behavioral choice in nematodes. For example, it has been shown that mutations of *glc-3* gene encoding a _L_-glutamate-gated chloride channel caused more nematodes to cross the Cu^2+^ barrier in the behavioral choice assay system [9].

Considering the fact that D1-like dopamine receptors can activate the Gα_q_ signaling pathway, and D2-like dopamine receptors can activate the Gα_o_ signaling pathway in cells of nematodes [2], we examined the roles of genes encoding Gα_q_ signaling pathway or Gα_o_ signaling pathway in regulating the behavioral choice. Our data suggest that, in the Gα_q_ signaling pathway, EGL-30 and EAT-16 regulated the behavioral choice of nematodes. In *C. elegans*, *egl-30* encodes the Gα_q_, and *eat-16* encodes the regulator of G protein signaling (RGS) protein, a GTPase activating protein. In the Gα_o_ signaling pathway, GOA-1 and EGL-10 regulated the behavioral choice of nematodes. In *C. elegans*, *goa-1* encodes the Gα_o_, and *egl-10* also encodes an RGS protein. These results suggest that both the Gα_q_ signaling pathway and the Gα_o_ signaling pathway are involved in the control of behavioral choice in nematodes.

**Figure 8:**
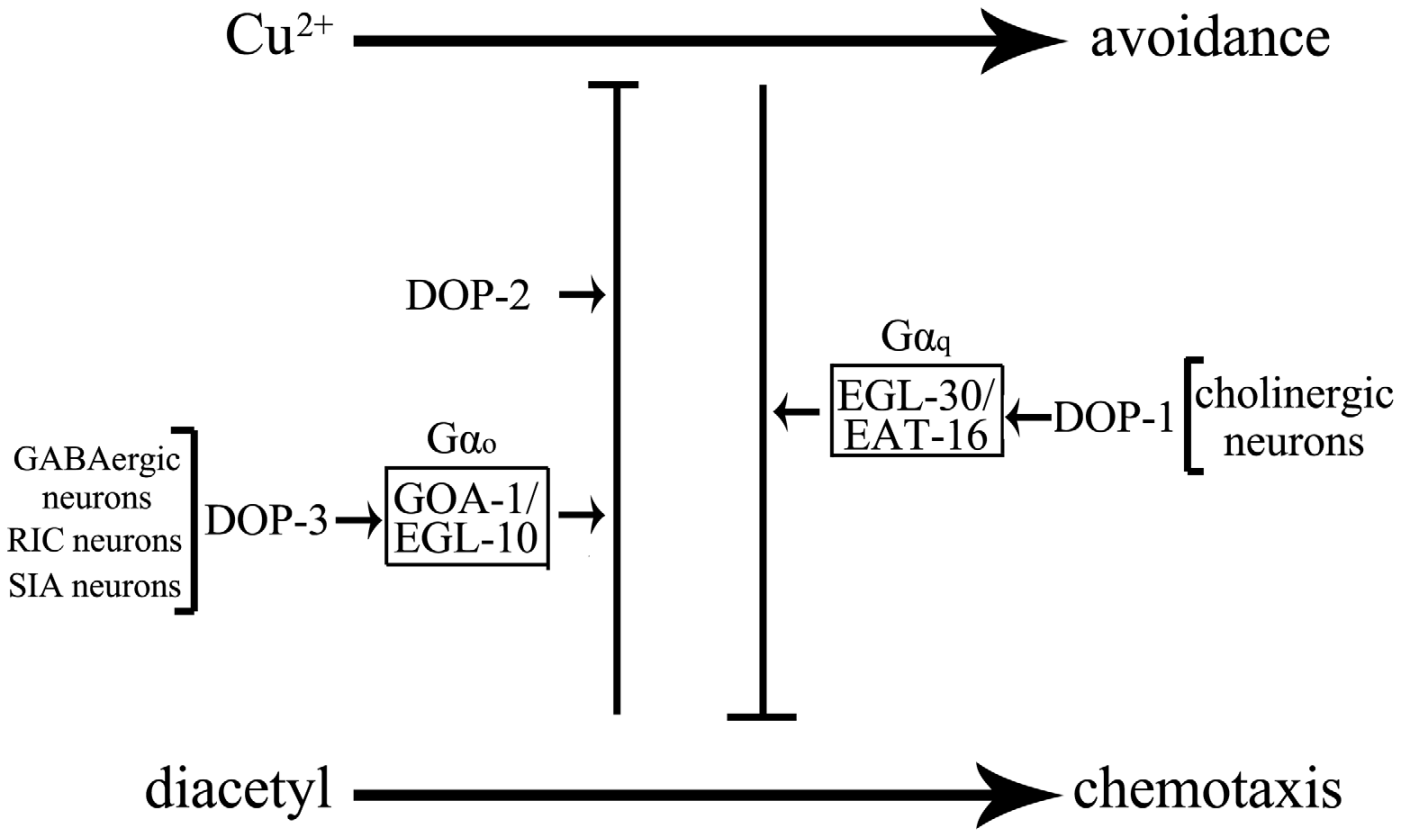
Model for dopamine receptors in regulating behavioral choice between conflicting alternatives in nematodes.

Moreover, the genetic assay indicate that DOP-1 could function in the same genetic pathway with EGL-30 and EAT-16 in regulating the behavioral choice, and DOP-3 could function in the same genetic pathway with GOA-1 and EGL-10 in regulating the behavioral choice. In this study, we hypothesize that D2-like dopamine receptor/DOP-3 acting through the Gα_o_ signaling pathway encoded by GOA-1 and EGL-10 may antagonize the function of D1-like dopamine receptor/DOP-1 acting through the Gα_q_ signaling pathway encoded by EGL-30 and EAT-16 to regulate the behavioral choice in nematodes (Fig. 8).

*C. elegans* is very suitable for understanding the neural signaling because of its simple and well-described nervous system [34]. Previous study has suggested that dopamine can counteract octopamine signaling in a neural circuit to regulate the food response [21]. Our work further revealed the neural circuit for D1-like dopamine receptor/DOP-1 or D2-like dopamine receptor/DOP-3 in regulating the behavioral choice in nematodes. Our data suggest that DOP-1 may function in cholinergic neurons to regulate the behavioral choice, and DOP-3 may function in GABAergic neurons to regulate the behavioral choice (Fig. 7). Nevertheless, the exact cholinergic or GABAergic neuron(s) in which DOP-1 or DOP-3 may function to regulate the behavioral choice is still unclear. Moreover, DOP-3 could further function in RIC or SIA neurons to regulate the behavioral choice (Fig. 7B). That is, DOP-1 and DOP-3 may function in different neurons to exert antagonistic effects on behavioral choice of nematodes (Fig. 8). Previous study has suggested that EGL-10, EAT-16, GOA-1, and EGL-30 can modulate the response of ASH sensory neurons to repellents in *C. elegans* [35]. Our results imply that DOP-1 and DOP-3 may only indirectly influence the function of ASH sensory neurons in regulating the response to repellents in nematodes. Previous study has also demonstrated that although DOP-3 is not expressed in ASH sensory neurons, *dop-3* RNAi knockdown in ASH casued the octanol hypersensitivity [36]. Besides these, previous study has demonstrated that adenosine A2A receptor antagonism can attenuate the effects from dopamine D2 antagonism on decision-making in mammals [16]. The possible interactions of dopamine signaling with other signaling pathways in regulating the behavioral choice are also needed to be further investigated.

In conclusion, in the present study, our data suggest that both D1-like and D2-like dopamine receptors can modulate the behavioral choice between conflicting alternatives (diacetyl and Cu^2+^). During the behavioral choice control, D2-like dopamine receptor DOP-3 could antagonistically regulate the function of D1-like dopamine receptor DOP-1 in nematodes. The behavioral choice phenotype of *dop-2; dop-1dop-3* triple mutant was similar to that in the single mutant of *dop-2* or *dop-3*. In nematodes, DOP-3 might act through the Gα_o_ signaling pathway encoded by GOA-1 and EGL-10, and DOP-1 might act through the Gα_q_ signaling pathway encoded by EGL-30 and EAT-16 to regulate the behavioral choice. Therefore, we here provide the direct genetic evidence to indicate the antagonistic relationship between D1-like dopamine receptor and D2-like dopamine receptor in regulating the decision-making of animals. In human beings, defects in dopamine signaling may underlie the neuronal development related diseases such as schizophrenia and autism [37]–[39]. Our data will be helpful for understanding the complex functions of dopamine receptors in regulating behaviors such as decision-making in animals.

## Materials and Methods

### Strains and genetics

The strains of wild-type Bristol N2, *dop-1(vs100)*X, *dop-2(vs105)*V, *dop-3(vs106)*X, *dop-4(ok1321)*X, *egl-30(n686)*I, *eat-16(ad702)*I, *egl-8(md1971)*V, *gpb-2(sa603)*I, *goa-1(sa734)*I, *egl-10(md176)*V, and *dgk-1(sy428)*X were originally obtained from the Caenorhabditis Genetics Center (funded by the NIH National Center for Research Resource, USA). *dop-1(vs100)*, *dop-2(vs105)*, *dop-3(vs106)*, and *dop-4(ok1321)* mutants are all animals with deletion mutations [2]. Nematodes were grown on nematode growth medium (NGM) plates seeded with Escherichia coli OP50 at 22°C as previously described [40]. Double mutant strains without additional marker mutations were constructed using standard genetic methods and verified by complementation testing.

### Behavioral assays

Body bend was counted for 1 min. A body bend was defined as a change in the direction of the part of the nematodes corresponding to the posterior bulb of the pharynx along the *y* axis, assuming that the nematode was traveling along the *x* axis. Fifty nematodes were examined per treatment, and five replicates were performed.

The method for assay for behavioral choice between conflicting alternatives (an olfactory (diacetyl) and a gustatory (metal ion) stimuli) was performed as basically described [7], [9], [12]. Twenty-five microliters of copper acetate solution was spread on the middle of the 9-cm assay plates (10 mM HEPES [Ph 7.0], 1 mM MgSO_4_, 1 mM CaCl_2_, 50 mM NaCl, and 2% agar), which were placed at room temperature for 20-h to allow the diffusion before assay. Fifty nematodes were placed on one side of the metal barrier on the assay plate, and 2 µL diluted diacetyl (10^−4^–10^−2^) was spotted on the other side. After 90 min, the numbers of nematodes on the original side [B] and on the odorant side [A] were scored. The index was calculated as A/(A+B) ×100(%). The behavioral changes after starvation was analyzed using young adults starved on NGM plates without bacteria for 5-h. Ten replicates were performed.

### DNA constructs and germline transformation

To generate entry vectors carrying promoter sequences, the promoter regions were amplified by PCR from *C. elegans* genomic DNA (2.0 kb for *hlh-17* promoter used for glia-specific expression, 0.9 kb for *unc-47* promoter used for GABAergic neurons-specific expression, 3.2 kb for *acr-2* promoter used for cholinergic neuron-specific expression, 2.8 kb for *gcy-28.d* promoter used for AIA-specific expression, 0.9 kb for *ttx-3* promoter used for AIY-specific expression, 3.0 kb for *tbh-1* promoter used for RIC-specific expression, 1.4 kb for *gcy-7* promoter used for ASE-specific expression, 2.3 kb for *sro-1* promoter used for expression in SIA neurons, and 1.2 for *lim-6* promoter used for expression in RIS neurons. The designed promoter primers were shown in S1 Table. And then these promoters were inserted into the pPD95_77 vector in the sense orientation. *dop-1* (the isoform of *F15A8.5a*), or *dop-3* (the isoform of *T14E8.3b*) cDNA was amplified by PCR. The sequences of the amplified cDNA were verified by sequencing, and then the cDNA was inserted into corresponding entry vectors carrying the appropriate promoter sequence. Germline transformation was performed as described [41] by coinjecting the testing DNA at a concentration of 10–40 µg/mL and the marker DNA of P*dop-1::rfp* at a concentration of 60 µg/mL into the gonad of nematodes.

### Statistical analysis

All data in this article were expressed as means ± standard error of the mean (S.E.M.). Graphs were generated using Microsoft Excel (Microsoft Corp., Redmond, WA). Statistical analysis was performed using SPSS 12.0 (SPSS Inc., Chicago, USA). Differences between groups were determined using analysis of variance (ANOVA). If not specifically indicated, the differences between groups were determined using one-way ANOVA. The probability levels of 0.05 and 0.01 were considered statistically significant.

## Supporting Information

S1 Table **Information for the designed promoter primers.**
(DOC)
