# Supplementary material for: Dopamine Receptors Antagonistically Regulate Behavioral Choice between Conflicting Alternatives in C. elegans
Source: PLoS One. 2014 Dec 23;9(12):e115985. doi: 10.1371/journal.pone.0115985 (PMC4275273; doi:10.1371/journal.pone.0115985)
Supplement: S1 Table — Information for the designed promoter primers. (DOC) [file pone.0115985.s001.doc]

**Table S 1. Information for the designed promoter primers.**

| Gene | Forward primer | Reverse primer |
| --- | --- | --- |
| P*hlh-17* | CGTCTGCAGCTATCCGTTTTCATTTCC | ATAGGATCCAAAACTCCGCCTCCAAAG |
| P*unc-47* | ATACTGCAGAAAGTAAGCAAATTCTAT | ATAGGATCCCATCTGTAATGAAATAAA |
| P*acr-2* | ATACTGCAGAAGTCAGAGCTGTGGAAG | ATAGGATCCGTCTTCTTCATGAAAACG |
| P*ttx-3* | TAATCTAGAATATGCACCCCGCTGACA | TTAGGATCCTTGTATTGACAGAATCCA |
| P*gcy-28.d* | CGCCTGCAGTACAATTGTAGTGAGCTT | ATAGGATCCTTCGCACTCATCTCACCA |
| P*lim-6* | ATTCTGCAGTTATTTCTTCCGAGTTAC | ATAGGATCCGTAGTAGTCTAGCACAAT |
| P*tbh-1* | CGGAAGCTTAGGATTCATCTACATTGC | ATAGGATCCCATTTTTCTGAAATCGTA |
| P*sro-1* | ATACTGCAGCCAAACAACTTCTAAGCT | ATAGGATCCCATTTTGTTTCGAAACTT |
| P*gcy-7* | ATACTGCAGAGACAGAAATAGGGGAAG | ATAGGATCCGGCTTCATGATTATTTTC |
